# Validity of Research-Grade Actigraphy Unit for Measuring Exercise Intensity

**DOI:** 10.3390/ijerph14050511

**Published:** 2017-05-10

**Authors:** Ke-Tsung Han, Po-Ching Wang

**Affiliations:** 1 Department of Landscape Architecture, National Chin-Yi University of Technology, 57 Sec. 2, Zhongshan Rd., Taichung City 41170, Taiwan; kthan@ncut.edu.tw; 2 Department of Landscape Architecture, National Chiayi University, 300 Syuefu Rd., Chiayi City 60004, Taiwan

**Keywords:** active living, energy expenditure (EE), metabolic heat (MH), physical activity (PA), sedentary lifestyle

## Abstract

This study was conducted in a free-living setting to investigate the measurement validity of a research-based actigraph for strolling and jogging, and to provide a reference for actual practice and research. Because inadequate physical activity (PA) or sedentary lifestyle has become the fourth leading risk factor for mortality worldwide, many countries have been vigorously promoting the concept of “active living”, and the public has been investing greater effort into intensifying their PA. Although research-grade actigraphs have been widely applied to evaluate PA in routine environments, the measurement results may not accurately reflect the wearers’ PA. Unlike most relevant research, which is conducted in well-controlled laboratory environments, the present study was implemented in the field to examine the sensitivity and convergent validity of the MicroMini Motionlogger^®^ Actigraph during strolling and jogging. The following results were revealed: (1) Although the exercise movement speed while jogging was significantly faster than that while strolling, the actigraph readings showed no significant difference between strolling and jogging; (2) The actigraph readings were (significantly or nonsignificantly) negatively correlated with metabolic heat and nonsignificantly correlated with movement speeds. Hence, the actigraph validity for measuring PA intensity while strolling and jogging remains debatable.

## 1. Introduction

The concept of “active living” is promoted in many countries to improve public health, as modern lifestyles are becoming increasingly sedentary with advances in society and technology [1,2,3]. During the past 50 years, the energy spent in daily activities by adults between 20 and 60 years of age in Europe and North America has dropped by 500 kcal [4]. In addition, according to a 2010 “Sport City Survey” conducted in Taiwan, up to 72.2% of people did not exercise regularly, which was higher than the 63.3% reported in the United Kingdom, 61.4% in Malaysia, 60.2% in Japan, 43.2% in the United States, and 32.5% in France [5]. According to statistics from the World Health Organization, approximately 60–85% of adults worldwide lead a sedentary lifestyle, and roughly 67% of children lack exercise. Moreover, physical inactivity or a sedentary lifestyle has become the fourth largest cause of death worldwide, and approximately 6% of annual mortality was found to be related to physical inactivity; more than two million deaths per year are attributable to a sedentary lifestyle [6]. In Taiwan, a recent study asserted that the intensity of physical activity (PA) was positively correlated with physical and mental health [7]. Consequently, active living has become an emerging trend with a philosophy based on exercise as a way of life and the recommendation of integrating PA into daily life.

With the growing interest in active living, a convenient method for facilitating objective and accurate gauging of PA has been much sought after. Although portable activity trackers may be useful for monitoring users’ physical behavior, the accuracy of these devices is arguable, and they could cause errors in the evaluation of daily exercise or in clinical judgment [8]. Presently, many people, including professional athletes, use wearable devices, mostly consumer-grade activity trackers, to record and appraise their PA. Among these devices, actigraphs are recognized as a method for the prolonged monitoring of PA, and they can objectively quantify frequency, intensity, and duration for assessment. Additionally, the output can also be cross-referenced with other activities that the wearer engages in [9,10,11,12,13]. Actigraphs, which are intended to be research-grade accelerometers, have been in use since the 1970s [14] and have been widely used in biomedical science [15], although their applications in sleep studies are considerably more prominent. Actigraphs generally have favorable reliability [16,17,18,19]. If an actigraph is consistently worn in the same place by the same user, it can achieve a reliability of 0.90–0.99 [20]; however, high reliability does not necessarily indicate an equally high validity. Reliability refers to the consistency of measures, whereas validity refers to the ability of measurements to approximate the actual state [21]. Recent studies have indicated that consumer-grade activity monitors may incorrectly quantify activity intensity [22]. In addition, consumer-level monitors may show only moderate convergent validity compared to research-grade devices, which are regarded as possessing acceptable accuracy for the measurement of PA and sleep time [23,24]. As the price of research-grade activity trackers decreases, more clinical professionals and the general public are using such units to monitor users’ daily exercise and light activity. Hence, this study was motivated by the need to examine the sensitivity and convergent validity of research-grade actigraph units. When a measure is highly correlated with other tests that are believed to measure the same construct, convergent evidence for validity is obtained. This type of evidence (for convergent validity) indicates that measures of the same construct converge [25].

One of the special features of the present study was the routine environment in which the experiment was performed: under daily living conditions instead of in a laboratory. This was intended to increase ecological validity. A systematic review of validity tests for activity trackers showed that only 3 out of 21 studies were conducted in the field [22]. In addition, in this study, the intensity of strolling and jogging, two of the most common PAs for leisure or exercise in Taiwan [26], was freely selected to closely reflect the lifestyles of the general public. In particular, most prior studies tended to recruit a limited number of participants because of the complexity of operating procedures under laboratory conditions [22]. However, in the present study, a relatively large number of subjects were recruited to improve the experimental validity. 

## 2. Literature Review

### 2.1. Actigraphy Devices

Actigraphy devices, or actigraphs, are usually worn on users’ nondominant arm or waist to gather movement data numerous times per second with built-in accelerometer or sensor units. These devices are sufficiently sensitive to measure the resultant force of body movements down to 0.01 g [27] and convert two- or three-dimensional movement data into analog waveform signals in minute-long epochs. Actigraphs can continuously measure and store movement data for up to one week [10]. Specialized computer software is then required to process the data and deduce the wearers’ active and static, awake and dormant, and acrophase patterns [16,17,18,19]. Before the activation of the device, a data acquisition mode, which cannot be changed during operation, must be selected. Two modes are available, namely the zero crossing mode (ZCM) and the proportional integrating measure mode (PIM). The ZCM measures the wearer’s movement frequency, which is represented by the number of times the voltage fluctuations of the analog signals exceed a predetermined threshold value. For example, when a swing-riding child starts swinging from a certain height, the ZCM will start counting the number of times the swing passes its equilibrium position (threshold), and continue counting until the swing slows down to a standstill. By contrast, the PIM records the fluctuation of analog signals when they exceed predetermined threshold values, using its own units (PIM counts). The absolute value or state of the waveform areas represents the intensity or extent of activity, ranging from 0 (i.e., a completely static body) to 32,000 (i.e., the body exercising at the highest intensity). For example, when two hammer-wielding individuals are subjected to a test of mechanical forces under identical conditions, the PIM will detect the variations in the strengths or forces they use. Therefore, the PIM concerns the measurement of intensity, instead of number of the times the activity exceeds a threshold value or a change in the activity pattern. Furthermore, the PIM can be categorized into low-PIM and high-PIM. Generally, low-PIM is ideal for monitoring premature infants or individuals with exercise-related disadvantages, whereas high-PIM is ideal for general users or individuals with hyperactivity disorder, because its specialized filters can amplify waveform signals for an improved comparison of activity intensity [28,29].

Actigraphs have been widely used in clinical medicine, particularly for monitoring the activities of patients, and they have had somewhat favorable results. Although actigraphs are recognized as effective data-gathering tools [30], their validity may be limited [31]. Recent studies have indicated that although the validity for step counting of consumer-grade activity trackers is acceptable [32], they may have lower validity for energy expenditure (EE) and sleep compared with other criterion measures [22]. Low accuracy in activity tracking might mislead and jeopardize professional clinical judgments, because these trackers are widely used as a reference for improving public health [8]. Research-based actigraphs are conventionally and widely used in medical evaluations of sleep and rest, such as the studies of circadian rest-activity cycles by Brown et al. [33] and wake-sleep conditions by Cole et al. [34] and Matsumoto et al. [35]. In addition, by studying 20 patients in intensive care units, Grap et al. [36] discovered that the readings of wrist actigraphs were significantly and positively correlated with the observed activity frequency, blood pressure, and mean arterial pressure of the patients. Rapport et al. [29] measured the activity intensity of 23 young boys by using low-PIM, and they found that actigraphs could assist in diagnosing attention deficit hyperactivity disorder. Moreover, Grap et al. [27] used actigraphs to assess the performances of 30 participants in a simulation of critically ill patients in calm, disturbed, and agitated states, which were also observed and recorded by accompanying nurses; the results revealed that both the activity records collected by observing nurses and the actigraphs in PIM mode could effectively distinguish the three states. Nevertheless, research-grade activity monitors may also have limited accuracy. A study that adapted direct calorimetry (e.g., room calorimeters) for criterion measurement revealed that even research-grade actigraphs, such as the ActiGraph GT3X, may not have ideal validity for EE [37]. Moreover, Abel et al. [38] recruited 20 participants who walked and ran for 10 min on treadmills at speeds of 54, 80, 107, 134, 161, and 188 m/min and found that EE was incorrectly measured by the ActiGraph GT1M at most walking and running speeds. Gusmer et al. [39] also demonstrated that the ActiGraph GT1M had poor-to-average agreement in the EE measurements of 21 participants who participated in two 30-min sessions of slow and brisk walking on treadmills. In addition, Bai et al. [31] indicated that the ActiGraph GT3X+ showed only 0.73 correlation with Oxycon Mobile (version 5.0) when testing EE during activities such as aerobic exercise, resistance exercise, and self-selected sedentary activity. 

### 2.2. Metabolic Heat (MH)

Metabolism involves the chemical reactions that transform consumed food into heat energy to sustain body functions. More specifically, the cells in the human body require certain types of chemical reactions to maintain a body temperature of 37 °C (98.6 °F). The amount of energy generated through “exercise activity thermogenesis” can be estimated from the metabolic rate and surface area of the human body. Therefore, the metabolic rate of a unit of body surface area varies with the intensity of activity the body engages in [40]. In both human physiology and medical science, body surface area (BSA) can be calculated using various formulas that differ only slightly with one another. The earliest such equation, proposed by DuBois et al. [41], is as follows:

BSA (m^2^) = 0.007184 × (W^0.4125^ × H^0.725^)

where W denotes body weight (kg) and H denotes height (cm). Although this formula is not suitable for infants, it is still the most widely used among researchers. Given that its error rate is lower than 5% when applied to varied ethnicities (e.g., Caucasian, Chinese, Indian, and Japanese) and body types [42], this formula was adopted in the present study for BSA calculation. Furthermore, MH was calculated as the product of BSA (m^2^) and metabolic rate (W/m^2^); metabolic rates are listed in Table 1 [40] and may be an alternative method for indirectly assessing the MH generated by strolling and jogging in the field. Estimating the MH through this approach is relatively inexpensive and easy to operate under free-living conditions.

Various techniques may also be adopted to indirectly evaluate metabolism. Traditionally, indirect calorimetry calculates metabolic rate by measuring oxygen consumption and carbon dioxide production to estimate heat production (HP), For example, for respirometry-based calorimetry gas collection and measurement equipment must generally be deployed around the subject under laboratory conditions, which makes the research process inconvenient. The doubly labeled water method, which is expensive but convenient, is another indirect method for estimating body metabolism using stable isotopes of hydrogen and oxygen [43]. By contrast, direct calorimetry quantifies temperature fluctuations in the HP of subjects through direct observation, generally by using a controlled chamber with subjects inside. Although direct calorimetry is the gold standard method for the accurate measurement of MH, its use is limited by high cost and inconvenience [44]. In general, most MH estimation methods conducted in laboratories, either through direct or indirect calorimetry, may not facilitate the research process because the operational procedure and mobility of the equipment are relatively limited. Although direct or indirect calorimetry testing under laboratory conditions might result in favorable internal validity, the ecological validity may be limited. Internal validity refers to whether the experimental treatments make a difference in the specific experimental instance, whereas ecological validity refers to samples of settings and participants that reflect the ecology of the treatments’ application [45].

The literature review on human body EE by Passmore et al. [46] proposed the following findings: walking speed was positively and linearly correlated with EE when the speed was 3–6.5 km/h; at 4.8 km/h, metabolism rate was independent of age, sex, or race; and at speeds greater than 7 km/h, EE grew exponentially with the increase in walking speed. When the ascending slopes featured gradients of 0%, 5%, 15%, and 25%, EE grew exponentially with the increase in gradient and walking speed; compared with walking on level ground, descending slopes consumed relatively less energy, whereas steep slopes consumed more energy. In addition, when running speed was 7–12 km/h, EE varied greatly. Another study used indirect calorimetry on 20 treadmill users engaged in walking (at 53.6, 80.4, and 107.2 m/min) and running (at 134.0, 160.8, 187.6, and 214.4 m/min) sessions that lasted 10 min at each speed with 2-min resting intervals, and found that the participants consumed significantly more energy as the treadmill speed was increased [47]. Recently, an indirect calorimetry study of 22 participants engaged in 5-min static-to-light activities in a laboratory found that, except for lying and sitting still and lying and standing still, all other activities consumed significantly different amounts of energy [48]. 

## 3. Methodology

### 3.1. Research Design

**Figure 1:**
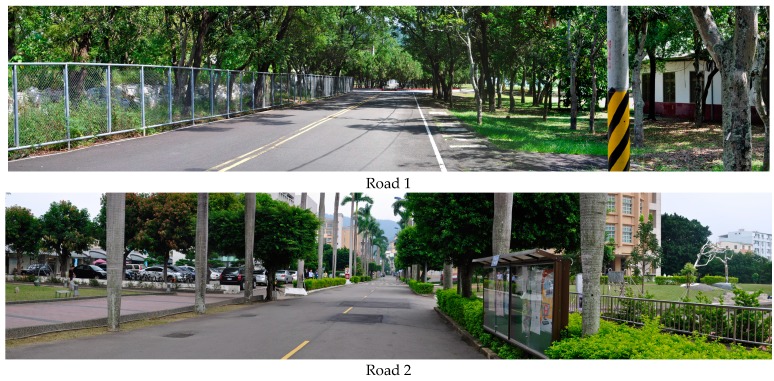
Experimental environment.

A major limitation of recent studies on the validity testing of actigraphs was that most evaluations of the intensity of daily PAs have been conducted in laboratories, whereas actigraphs are usually applied in free-living environments [8,22]. Because of the favorable ecological validity of field experiments [45], in the present study, a randomized controlled trial was conducted that assigned participants with different experimental treatments in actual environments. The experimental treatments comprised strolling and jogging sessions, which are the most popular exercises in Taiwan and worldwide [49]. The participants were asked to stroll or jog continuously for 15 min at their chosen speed back and forth along two 400 m, east-west parallel, level, and paved roads on the campus of National Chin-Yi University of Technology in central Taiwan (Figure 1). The participants were only permitted to break for safety reasons. The field experiment lasted for from October 2013 to February 2014, and all sessions took place between 8 am and 5 pm.

### 3.2. Instruments and Data Collection

BSA was calculated according to the formula of DuBois et al. [41] using participant data (e.g., height and body weight) obtained from a questionnaire. In addition to height and body weight, the questionnaire also inquired about background information such as health, physical fitness, sex, age, college attended, and year at school. The MicroMini Motionlogger^®^ Actigraph (Ambulatory Monitoring, Inc., New York, NY, USA), which has been applied in areas including sleep research, was used in this study. The actigraph was worn on the participants’ nondominant arm for objectively gauging and recording their activity intensities [9]. Because the participants were all ordinary college students, high-PIM was chosen as the most suitable measurement model. Moreover, movement speed was calculated by dividing movement distance with movement time. Each participant’s movement distance was measured by a research assistant, who closely followed the participant, by using the global positioning system (GPS) feature of the Nike+ mobile application (Nike, Inc., Beaverton, OR, USA). The Nike+ mobile application is popular among joggers and has an accuracy of ±5 m [50]. Although the real-time specific movement speeds of the participants and the research assistant may not have been exactly identical, their average movement speeds during the 15-min session were close. Furthermore, to determine the MH of each participant, corresponding metabolic rates were obtained from Table 1 [40] according to participants’ strolling or jogging speeds, which were then multiplied with BSAs.

### 3.3. Participants

Unlike most previous validity tests, which have involved a small number of participants [22], a relatively large number of participants were involved in this study. A total of 120 Han Chinese college students were recruited from National Chin-Yi University of Technology for this research. All participants provided informed consent. To ensure the participants’ safety, they were interviewed about their histories of physical injuries, asthma [51], and allergies to sunlight, air, or plants. Moreover, they were required to answer and pass the Physical Activity Readiness Questionnaire (PAR-Q) [52] before being included in the experiment. The PAR-Q, containing seven questions, is designed to assess the fitness of those aged 15–69 years who intend to take up exercise or intensify their PA. The PAR-Q was adopted to determine whether the prospective participants were in a sufficiently physically fit condition to safely participate in the experiment. All prospective participants passed the PAR-Q.

## 4. Results

### 4.1. Descriptive Statistics of Sample Distribution

After the exclusion of invalid participants who did not complete the experiment, 116 participants remained (52 males, 64 females). Among them, 29 participants strolled and 29 jogged on Road 1, and 32 strolled and 26 jogged on Road 2. The average age of these valid participants was 20.85 years (SD = 1.14). Their average weight, height, and BMI (body mass index) were 58.14 kg (SD = 10.93), 165.55 cm (SD = 8.57), and 21.14 kg/m^2^ (SD = 3.11), respectively. Table 2 lists additional background details. The results of statistical analyses showed no significant differences by sex (χ^2^ = 1.625, *p* = 0.654); college attended (χ^2^ = 9.555, *p* = 0.145); academic year (χ^2^ = 6.556, *p* = 0.885); age, height, and weight (Box’s M = 29.399, *F* = 1.553, *p* = 0.063; Pillai’s Trace ≤ 0.041, *F*_(3,110)_ ≤ 1.563, *p* ≥ 0.202, $\eta_{p}^{2}$ ≤ 0.041); or BMI (*F*_(__1,112__)_ = 3.728, *p* = 0.056, $\eta_{p}^{2}$ = 0.032) with respect to exercise groups and exercise roads.

### 4.2. Analyses of Variance

Because the participants were permitted to stroll or jog at their chosen speed, descriptive statistics of these speeds, which were measured using the GPS tracking distances divided by the 15-min exercise duration, are presented first in Table 3.

**Figure 2:**
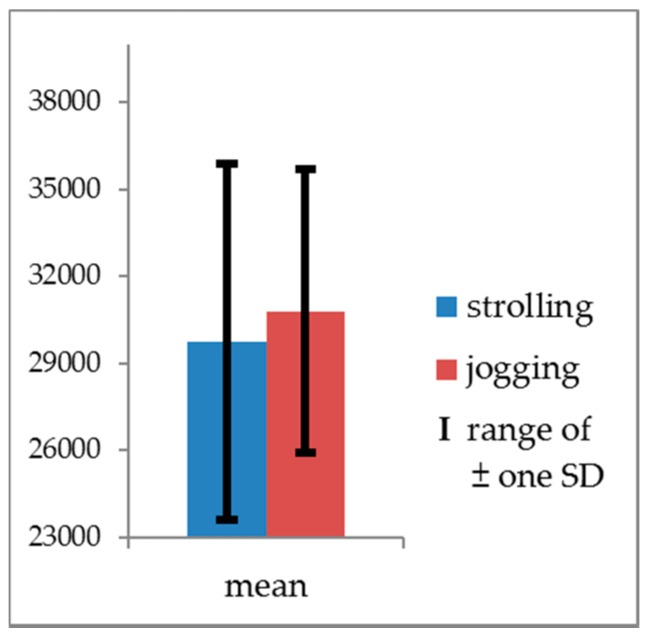
Means and SDs of actigraph readings (PIM counts).

**Figure 3:**
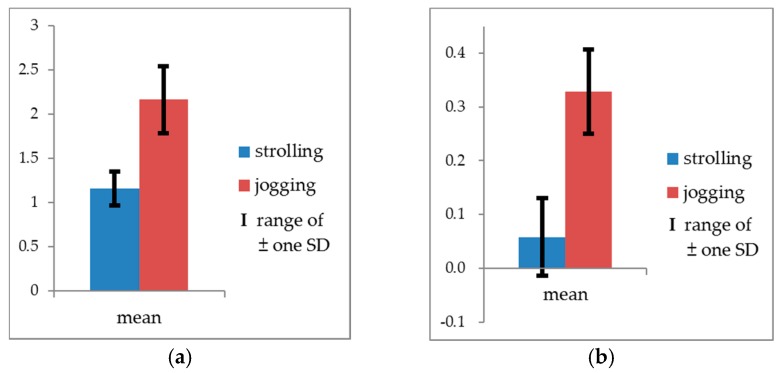
Means and SDs of movement speeds (m/s). (**a**) Raw data; (**b**) Logarithmic transformations.

Additionally, to verify the effectiveness and sensitivity of the measurements in distinguishing the exercise intensity of strolling and jogging, one-way multivariate analysis of variance (MANOVA) was employed to identify differences between the actigraph readings and the movement speeds. Because the movement speeds in this experiment violated the assumption of homogeneity of variance, and the raw data values were all >0, the raw data underwent logarithmic transformation before being examined. The transformed data structure was not found to violate the assumption of homogeneity of variance required for MANOVA (Box’s M = 3.666, *F* = 1.199, *p* = 0.309). Given the unequal sample sizes in the strolling and jogging groups, Pillai’s Trace (*V*) was used in the overall test. The result was significant (Pillai’s Trace = 0.771, *F* = 190.257, *p* < 0.001). Subsequently, one-way analysis of variance (ANOVA) was used for the follow-up test. Because there were two dependent variables, a correction was necessary for the significance level of ANOVAs (α = 0.05/2 = 0.025) to avoid family-wise errors (FWE) or cumulative Type I errors [53]. According to the tests, strolling and jogging exhibited no significant difference in actigraph records, but they achieved significant difference in movement speeds (*F_(_*_1,114)_ = 376.635, *p* < 0.001) with a high effect size ($\eta_{p}^{2}$ = 0.768) [54]. The jogging speeds were significantly greater than those of strolling (Table 4, Figure 2 and Figure 3). Consequently, the manipulation of this experiment was deemed valid. 

### 4.3. Correlation Analysis

**Figure 4:**
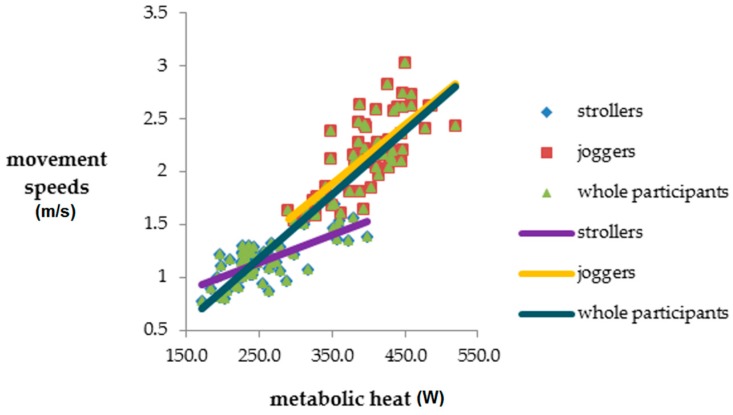
Correlations between metabolic heat (MH) and movement speeds among strollers, joggers, and all participants.

Table 5 shows the correlation analysis results for the strollers, the joggers, and all participants with respect to actigraph readings, movement speeds, and MH. The results indicated that the correlations between movement speeds and actigraph readings were nonsignificant (all *p* > 0.05) for the strollers, the joggers, and all participants. Moreover, actigraph readings were found to be negatively correlated with MH among the strollers, the joggers, and all participants; however, this correlation reached significance only with the strollers (*p* = 0.015). Meanwhile, movement speeds and MH exhibited a highly positive correlation (all *p* < 0.001), for the strollers, the joggers, and all participants (Figure 4).

## 5. Discussion

The field experiment on the validity of actigraphs to gauge the activity intensities of strolling and jogging revealed the following findings: (1) the jogging speeds determined by the participants were significantly higher than the participant-determined strolling speeds, with a high effect size; (2) the actigraph readings exhibited no significant difference between strolling and jogging, which was considered a sign of low sensitivity; (3) movement speeds and actigraph readings had a weak and nonsignificant correlation for strollers, joggers, and all participants, which indicated low convergent validity; (4) the actigraph readings exhibited negative correlations (either significant or nonsignificant) with MH, which again indicated low convergent validity; (5) movement speeds were significantly and positively correlated with MH for strollers, joggers, and particularly for all participants; and (6) given the negative correlations between movement speeds and actigraph readings and the negative correlation between MH and actigraph readings for strolling, actigraphs appeared to be particularly inappropriate for measuring low-intensity activities, which is in agreement with the findings of similar studies [38,39]. 

The strong positive correlations between movement speeds and MH were not surprising, because the metabolic rates that were used to calculate MH rose with movement speed [40]. Because MH accounts for not only metabolic rates related to movement speeds but also individuals’ heights and weights (i.e., BSAs), it may provide additional information and different perspectives on activity intensities other than movement speeds. This is illustrated by the different correlations between MH and movement speeds among strollers, joggers, and all participants. If MH and movement speeds were equivalent, they would have had a perfect correlation coefficient of 1. In addition to movement speeds and MH, future investigations regarding the validity of actigraph are suggested to introduce other criteria that are (1) not correlated with movement speeds and (2) present continuous and precise values, unlike the metabolic rates in Table 1, which are simply representative values for speed intervals. Furthermore, direct or indirect calorimetry devices are ideal for laboratory settings but are relatively infeasible for application in free-living environments. The activity intensities of the participants can also be measured using other approaches that are more suitable for field studies. For example, physiologic monitors can gauge heart rate, which is correlated with activity intensity, and blood pressure [55,56]. In addition, the EE of bodily movements can be estimated using metabolic equivalent task (MET) minutes [57]; the EE is calculated as the minutes engaged in an activity multiplied by the specific numbers of MET minutes required for that specific activity. Moreover, measuring activity intensity is not necessarily limited to EE. The Intelligent Device for Energy Expenditure and Activity (MiniSun LLC, Fresno, CA, USA) can measure not only EE but also limb movements, limb positions, change of positions, and gaits [58], which may provide deeper insights into PA. 

This field experiment adopted a between-subjects design with a randomized controlled trial. Statistical analyses revealed no significant differences between the backgrounds of the participant groups. Given that all participants were Han Chinese, the possible bias due to phenotypical traits between groups was limited. Nevertheless, the results may not be generalizable to other ethnic groups. Alternatively, this study could have adopted a within-subjects design to account for the variability in the groups assigned to strolling and jogging. For such a design, all participants would have needed to both stroll and jog. Even with breaks between strolling and jogging, results without confounding effects between these two PAs would not have been guaranteed. Because PA is related to physical fitness [59], a PA conducted later may be influenced by a PA conducted earlier in the day, particularly among the general population, whose physical fitness may be lower than that of athletes [38].

The fact that participants’ heights and weights were self-reported rather than measured was a shortcoming of this study. The negative but nonsignificant correlations between actigraph readings and MH may have been because the self-reported heights and weights were inaccurate, which resulted in incorrectly estimated MH, or because the actigraph was inappropriate for measuring the EE. 

The implementation of the experiment could also be improved in future studies. The participants were required to wear the actigraph on their nondominant arm in the present study, which might not swing at a significantly different amplitude or speed during strolling and jogging. Therefore, future investigators are suggested to place the device both on the participants’ wrists and ankles to further verify its validity [27]. Furthermore, the GPS device was carried by the assistants in the present study. This practice may be replaced with the participants wearing a GPS device on their wrist [55] that is supported by geographic information system software. This is expected to improve the accuracy of measurement for movement across undulating terrain, because this technology can provide accurate and prolonged monitoring of three-dimensional positioning and movement duration. This will further improve the measurement of EE, which varies with terrain [46]. These issues warrant further investigation.

## 6. Conclusions

Research-grade actigraphy is conventionally regarded as an effective method for assisting the medical evaluations of subjects’ PA [30]. However, in the present field experiment, the MicroMini Motionlogger^®^ Actigraph was found to be insufficiently sensitive to distinguishing the PA intensities of strolling and jogging, despite the difference in movement speeds. Further studies are needed to examine whether actigraphy is an appropriate measurement method for the activity intensities of specific exercises, particularly in combination with other criterion measures. Because sedentary lifestyles have become the world’s fourth leading cause of death, PA is a matter of public health and well-being; consumer- and research-grade devices for the convenient and objective measurement of activity intensity are instrumental to the promotion of active living.

## Figures and Tables

**Table 1:** Metabolic rates.

Movement Speed (m/s)	Metabolic Rate (W/m^2^)	Note
0.89	115	115 W/m^2^ for <0.89 m/s
1.34	150	150 W/m^2^ for 0.90–1.34 m/s
1.79	220	220 W/m^2^ for 1.35–1.79 m/s
1.80	255	255 W/m^2^ for >1.80 m/s

Source: Kreider et al. [40].

**Table 2:** Descriptive statistics of participants.

Background	Classification	Number of Participants (*n* = 116)	Percentage (%)
Sex	Male	52	44.8
	Female	64	55.2
Age	18–20 years	45	38.8
	21–24 years	70	60.3
	25–30 years	1	0.9
	>31 years	0	0
Height	141–150 cm	6	5.2
	151–160 cm	29	24.9
	161–170 cm	48	42.3
	171–180 cm	27	23.2
	>181 cm	5	4.4
Weight	40–50 kg	26	22.4
	51–60 kg	56	48.1
	61–70 kg	24	20.7
	71–80 kg	7	6.1
	81–90 kg	2	1.8
	>91 kg	1	0.9
College	Engineering	0	0
	Management	5	4.3
	Electrical engineering & computer science	27	23.3
	Humanities and creativity	84	72.4
Year	Freshman	17	14.6
	Sophomore	43	37.1
	Junior	50	43.1
	Senior	5	4.3
	Graduate	1	0.9

**Table 3:** Descriptive statistics of movement speeds.

Exercise Type	Number of Participants	Minimum (m/s)	Maximum (m/s)	Mean (m/s)	Standard Deviation (m/s)	Skewness	Kurtosis
Strolling	61	0.77	1.69	1.16	0.19	0.29	0.46
Jogging	55	1.54	3.03	2.17	0.38	0.04	−0.85

**Table 4:** Statistical results for jogging and strolling.

Dependent Variable	MANOVA (Overall Test)			ANOVA (Follow-Up Tests)			
	*F*	*p*	Pillai’s Trace (*V*)	*F*	*p*	Partial Eta Squared ($\eta_{p}^{2}$)	Post-Hoc Tests
Actigraph readings	190.257 ***	<0.001	0.771	0.995	0.321	0.009	N/A
Movement speeds				376.635 ***	<0.001	0.768	Jogging > Strolling

Notes: Independent variable = Exercise type; FWE = 0.025 (Bonferroni approach); *** denotes *p* < 0.001.

**Table 5:** Results of correlation analysis with Pearson’s correlation coefficient.

Variable	Actigraph Readings			Movement Speeds		
	Strollers	Joggers	Whole	Strollers	Joggers	Whole
	(*n* = 61)	(*n* = 55)	(*n* = 116)	(*n* = 61)	(*n* = 55)	(*n* = 116)
Movement speeds	−0.179	0.002	0.047	N/A	N/A	N/A
	(*p* = 0.167)	(*p* = 0.990)	(*p* = 0.614)			
MH	−0.310 *	−0.009	−0.032	0.714 ***	0.748 ***	0.905 ***
	(*p* = 0.015)	(*p* = 0.950)	(*p* = 0.733)	(*p* = 0.000)	(*p* = 0.000)	(*p* = 0.000)

Notes: two-tailed tests, where * denotes *p* < 0.05 and *** denotes *p* < 0.001.
